# Annual Meeting of Military Tract Med. Association, at Galesburg, Ill.

**Published:** 1872-07

**Authors:** 


### Proceedings of Societies.

*Annual Meeting of the Military Tract Medical Association, at Galesburg, Ill., June* 11, 1872.

Pursuant to adjournment, the Seventh Annual Meeting of the Military Tract Medical Association convened at Masonic Hall, in Galesburg, June 11th, at 10 o'clock, A. M. In the absence of the President, Dr. Phillips, of Galesburg, was called to the chair. The Board of Censors being absent, Drs. Reece, McClelland and Klingberg were appointed. The board then presented the names of Drs. Bunce, Heller, D. T. Brown and Sapp for membership. The association received them by a unanimous vote.

The regular order of business, the election of officers for the ensuing year, was laid over until the afternoon session. Dr. W. L. Cuthbert then offered a series of resolutions concerning abortion, which after some discussion were referred to a committee consisting of Drs. Cuthbert, Reece, Hurd, Bacon and Bunce; upon retirement of the committee the association adjourned until 2 o'clock.

AFTERNOON SESSION.

The association re-assembled at 2 o'clock, with Dr. Phillips in the chair. On motion, the association proceeded to the annual election of officers, with the following result:

John W. Hensley, of Yates City, President.
M. A. McClelland, of Knoxville, Vice-President.
Dr. Herbert Judd, of Galesburg, Secretary and Treasurer.

Dr. Hensley returned thanks on taking the chair, in a brief address, promising to discharge the duties to the best of his ability. He then called for the reports of the committees, when Dr. Reece, chairman of the committee to whom Dr. Cuthbert's resolutions were referred, made a favorable report.

On motion, the report was received and the committee discharged. These resolutions are of the greatest interest, and are as follows:

WHEREAS, The procuring of criminal abortion is becoming more prevalent, and in our vicinity as elsewhere, unscrupulous specimens of human depravity—abortionists—are plying their murderous traffic; and that from the members of the medical profession the public expect an expression of their opinion in regard to this evil; therefore,

*Resolved,* That we recognize the procuring of miscarriage or abortion in any stage of pregnancy as criminal in the highest degree, unless absolutely demanded to save human life, and then only by the advice of two or more regular physicians.

*Resolved,* That we, as physicians, pledge all our efforts to assist and sustain our legislators and executives in all measures that tend to the suppression and prevention of this heinous crime, with its fearful consequences.

*Resolved,* That we are bound by a sense of duty as physicians and citizens to condemn the abortionist and his abettors, deeming them unworthy of our association and respect; and to use all reasonable means to expose their murderous schemes and bring them to punishment.

*Resolved,* That while we denounce their murderous traffic we will strive to inform the public of its train of consequences; for the violation of physical and moral laws is certain to meet its just penalty—prolonged and intractable diseases of body and mind, and often death.

*Resolved,* That a copy of this preamble and resolutions be forwarded to each paper published within our bounds, the *Chicago Medical Journal* and *Chicago Medical Examiner* inclusive, with a request for gratuitous publication, that the position of the Military Tract Medical Association may be known on this matter of vital importance to the public.

From the Standing Committees on Surgery, Practice of Medicine, Materia Medica, Obstetrics and Diseases of Women and Children, many valuable and interesting reports were made, both written and verbal. More time was spent in discussion than at any previous meeting.

Under Miscellaneous Business, Drs. Reece, Judd, Phillips, McClelland, and Webster, were appointed a committee to revise the constitution and by-laws.

Drs. Phillips, Morse and Hurd were appointed a committee to report at the next meeting upon the death of Dr. John W. Spalding.

The following are the Standing Committees, to report at the next meeting to be held in January:

*Censors*—M. Reece, M. A. McClelland, A. Klingberg.

*Essayists*—W. L. Cuthbert, J. R. Webster.

*Surgery*—M. A. McClelland, J. M. Morse, Wm. Hamilton.

*Practice of Medicine*—E. L. Phillips, B. F. Brown, J. K. Seacord.

*Materia Medica*—Henry B. Upton, D. W. C. Bacon, Charles Bunce.

*Obstetrics, and Diseases of Women and Children*—W. H. Heller, D. T Brown, T. J. Maxwell.

*Ophthalmology*—L. S. Lambert.

Upon motion, the association adjourned, to meet in Galesburg on the second Tuesday in January next.

